# Bone Mineral Density in HIV-Infected People–the Experience of Craiova Regional Center

**DOI:** 10.3390/biomedicines13092305

**Published:** 2025-09-20

**Authors:** Florentina Dumitrescu, Livia Dragonu, Eugenia-Andreea Marcu, Vlad Pădureanu, Andreea Cristina Stoian, Cristiana-Luiza Rădoi-Troacă, Rodica Pădureanu, Anca Duduveche, Ilona-Andreea Georgescu, Lucian Giubelan

**Affiliations:** 1 Department of Infectious Disease, University of Medicine and Pharmacy of Craiova, 200349 Craiova, Romania; florentina.dumitrescu@umfcv.ro (F.D.); livia.dragonu@umfcv.ro (L.D.); andreea_plr@yahoo.com (A.C.S.); luizacristianaradoi@gmail.com (C.-L.R.-T.); anca.duduveche@umfcv.ro (A.D.); georgescuilonaa@gmail.com (I.-A.G.); lucian.giubelan@umfcv.ro (L.G.); 2 ”Victor Babes” Hospital of Infectious Diseases and Pulmonology from Craiova, 200515 Craiova, Romania; 3 Department of Internal Medicine, University of Medicine and Pharmacy of Craiova, 200349 Craiova, Romania; rodica.padureanu@umfcv.ro

**Keywords:** antiretroviral, bone mineral density, HIV-related comorbidities, osteoporosis, osteopenia

## Abstract

**Background:** Human Immunodeficiency Virus (HIV) is a virus that progressively impairs immune function by depleting CD4 + T-lymphocytes, ultimately leading to acquired immunodeficiency syndrome (AIDS). People living with HIV face a higher risk of developing various bone disorders, such as osteopenia, osteoporosis, and osteonecrosis. The aim of this study was to evaluate the bone mineral density (BMD) status, to determine the prevalence of osteopenia/osteoporosis and to identify the risk factors for low BMD in patients living with HIV undergoing antiretroviral treatment (ART), registered in Craiova Regional Center. **Methods:** A retrospective study was conducted between June 2024 and January 2025, including HIV-infected subjects aged over 18 years. **Results:** The study group included 106 patients. Dual-energy X-ray absorptiometry (DEXA) showed that 87 patients had low BMD, 51% having osteopenia and 31.1% having osteoporosis. We found a statistically significant correlation between low BMD and older age, higher levels HIV viremia, CD4 nadir < 200 cells/mm^3^, prolonged ART exposure and tenofovir disoproxil fumarate containing regimens. **Conclusions:** These findings support the inclusion of routine bone health monitoring in the standard care of patients with HIV, as well as the need for reevaluation.

## 1. Introduction

### 1.1. Epidemiology

According to the World Health Organization (WHO), at the end of 2023, an estimated 39.9 million people [36.1 million−44.6 million] were living with human immunodeficiency virus (HIV). In 2023, an estimated 1.3 million people [1.0 million−1.7 million] acquired HIV. Since 2010, the number of new HIV cases has decreased by 39%, from 2.1 million [1].

Since the introduction of antiretroviral therapy (ART), both life expectancy and quality of life have greatly improved for people living with HIV (PLHIV). In 2023, 77% [61–89%] of PLHIV were receiving ART [1].

Since 1985, Romania has recorded 28.793 cases of HIV/AIDS and 18.768 people were living with HIV/AIDS in Romania as of December 31 st 2024 [2]. There are specific epidemiological features associated with HIV infection in Romania. Between 1986 and 1991, many children in Romanian hospitals and orphanages were infected with HIV as a direct consequence of government policies that led to widespread exposure to contaminated needles and unscreened blood used in so-called microtransfusions [3,4].

### 1.2. Influence of HIV Infection and ART on Bone Health

Although ART has significantly improved survival and quality of life for PLHIV, metabolic complications such as cardiovascular diseases, dyslipidemia, decreased bone mineral density (BMD), fragile fractures, and insulin resistance are associated with HIV infection. These issues arise from factors including chronic inflammation, persistent HIV viremia, accelerated aging, and the long-term side effects of ART [5]. Highly active antiretroviral therapy (HAART) may act as a double-edged sword. It improves life expectancy in people with HIV/AIDS but may also trigger or worsen autoimmune diseases. While it restores immune function and may reduce autoimmune activity, it can also lead to new or reactivated autoimmune conditions through immune reconstitution inflammatory syndrome (IRIS), resembling a delayed hypersensitivity response [6].

PLHIV face a higher risk of developing various bone disorders, such as osteopenia, osteoporosis, and osteonecrosis. These conditions lead to an increased likelihood of both traumatic and non-traumatic fractures, affecting up to 7% of those affected [7,8]. Several factors contribute to this risk, including the higher occurrence of traditional bone disease risk factors in PLHIV, chronic inflammation, low body weight, hypogonadism, intravenous drug use, and co-infection with hepatitis C. [9].

Antiretroviral therapy has significantly improved survival in PLHIV, but it is also associated with early bone mineral density loss, particularly during the first years of treatment. Tenofovir disoproxil fumarate (TDF) and protease inhibitors (PIs) are the most strongly implicated, through mechanisms such as renal phosphate wasting, mitochondrial toxicity, and increased bone turnover, which predispose patients to premature osteopenia and osteoporosis. This emphasizes the need for early bone health monitoring in PLHIV receiving ART [10].

Some other factors have been identified as contributing to osteopenia in PLHIV. These include direct effects of the virus on osteogenic cells, ongoing activation of pro-inflammatory cytokines, particularly tumor necrosis factor alpha (TNFα) and interleukin−1, alterations in vitamin D metabolism leading to a deficiency in 1,25-dihydroxyvitamin D, and mitochondrial abnormalities associated with lactic acidosis and the onset of opportunistic infections [5].

HIV proteins, including gp120 and Tat, can directly interact with bone cells [11]:

- Osteoblasts: These bone-forming cells can be inhibited by HIV proteins, reducing bone formation.
- Osteoclasts: HIV can stimulate osteoclastogenesis, leading to increased bone resorption.

This imbalance between bone formation and resorption contributes to decreased BMD.

HIV infection can disrupt endocrine function, leading to: hypogonadism-reduced levels of sex hormones like testosterone and estrogen decrease bone formation and increase bone resorption and vitamin D Deficiency-common in HIV-positive individuals, vitamin D deficiency impairs calcium absorption, essential for bone mineralization [11].

Individuals with HIV often experience malnutrition (inadequate intake of essential nutrients weakens bone structure), low body weight (associated with lower bone mass), smoking and alcohol use (both being detrimental to bone health) [11].

Coinfection with hepatitis C virus and other chronic conditions can further increase the risk of osteoporosis and fractures in people living with HIV. The combined effects of these infections and associated treatments can exacerbate bone loss [11].

Age plays a significant role in HIV treatment outcomes and may contribute to senile osteoporosis. HIV proteins increase osteoclast activity and promote osteoblast apoptosis, which restricts bone production. Additionally, viral infection can affect BMD through systemic inflammation and bone remodeling [12]. Research supports these findings, showing a higher incidence of osteoporosis in PLHIV. Among those receiving highly active antiretroviral therapy, the prevalence of osteoporosis is nearly three times higher compared to other infected individuals [13].

The association between HIV infection and low BMD is multifactorial, involving direct viral effects on bone cells, chronic inflammation, ART side effects, hormonal imbalances, and lifestyle factors. Understanding these mechanisms is crucial for developing strategies to prevent and manage bone loss in HIV-infected individuals.

### 1.3. Diagnosis Criteria for Low BMD

The most recommended method for diagnosing osteoporosis is measuring BMD using dual-energy X-ray absorptiometry (DEXA), typically at two sites: the proximal femur and the lumbar spine. If these sites are unavailable for testing, the forearm (radius) is used as an alternative.

WHO defines osteopenia and osteoporosis based on bone densitometry results, where a patient’s bone density is compared to the average for young adults, with adjustments made for race and gender. Osteoporosis is diagnosed when the bone density is more than −2.5 standard deviations below the average and reflects a high risk of fracture, typically necessitating pharmacologic intervention, while osteopenia is diagnosed when the result falls between −2.5 and −1.0 standard deviations and corresponds to an intermediate fracture risk, in such cases, treatment decisions being often guided by additional clinical risk factors and individual patient context. A T-score of >−1.0 is considered normal and denotes a low risk of fracture, for which pharmacologic therapy is generally not required [14,15].

It is recommended to screen postmenopausal women and men over the age of 49 with DEXA scanning. Treatment involving calcium and vitamin D supplementation and/or bisphosphonates has proven to be both safe and effective for PLHIV. However, there is limited data available regarding the use of hormonal therapy in PLHIV [9].

### 1.4. Study Aim

In this context, the present study aimed to evaluate bone mineral density, determine the prevalence of osteopenia and osteoporosis, and identify clinical and treatment-related risk factors associated with low BMD in people living with HIV who are undergoing antiretroviral therapy and are registered at the Craiova Regional Center (CRC).

## 2. Materials and Methods

We performed a retrospective study between June 2024 and January 2025, which included HIV-infected subjects aged over 18 years, recorded in CRC. During the study period, 106 patients were evaluated.

### 2.1. Inclusion Criteria

- PLHIV on ART who were registered in our clinic and had not previously received calcium or vitamin D supplementation.
- Patients being on ART for at least 1 year.

### 2.2. Exclusion Criteria

- Endocrine disorders: hyperthyroidism, hyperparathyroidism, hypogonadism, diabetes mellitus, Cushing’s syndrome.
- Autoimmune diseases.
- Use of drugs that affect bone metabolism.
- Patients already undergoing treatment for osteoporosis.
- Patients with incomplete medical records.

In our study, we collected epidemiological and demographic data, data on gender, age, smoking and alcohol consumption, body mass index (BMI), 25-hydroxyvitamin D (25[OH]D) level, BMD measurement, CD4 count, HIV viral load (VL-HIV) level, data on ART, data on co-infections with hepatitis viruses.

Serum 25-hydroxyvitamin D [25(OH)D] levels were measured using a chemiluminescence immunoassay on the Alinity i system (Abbott Laboratories, Abbott Park, IL, USA), following the manufacturer’s instructions. The 25[OH]D deficiency was considered at a level below 20 ng/mL.

Body mass index was classified according to WHO guidelines. BMI < 18.5 kg/m^2^ was considered as underweight, 18.5–24.9 as normal weight, 25–29.9 as overweight, and 30≥ as obesity [16].

CD4 cell count was determined by flow cytometry.

Bone mineral density was assessed using DEXA technique at three sites: the distal forearm, lumbar spine (L1-L4), and femoral neck. DEXA was reported with T and Z-scores. The patients were classified as normal, osteopenic (T-score between −2.5 and −1), or osteoporotic (T-score below −2.5) based on DEXA measurements according to WHO criteria.

All data were collected using medical records of PLHIV, registered within CRC, as well as the electronic database. All analyses were performed by using Microsoft Excel version 2019 (Microsoft Corporation, Redmond, WA, USA) (Analysis ToolPak). For categorical data, Chi-square and Fisher’s exact tests were used, while Pearson correlation, T-test were applied for continuous variables. Logistic regression was performed to assess binary outcomes (osteopenia/osteoporosis vs. normal BMD), and linear regression was used for continuous dependent variables (e.g., T-scores). Multivariate regression analyses were adjusted for age, sex, BMI, vitamin D status, HIV viral load, and ART regimen. A two-tailed *p*-value < 0.05 was considered statistically significant.

We incorporated the results into charts and tables.

All the participants in the study signed an informed consent upon admission for processing of medical data.

This study was sponsored by the Medicina Foundation and was conducted in accordance with the guidelines of the Declaration of Helsinki. The research protocol was approved by the Ethics Committee of the University of Medicine and Pharmacy of Craiova, Romania (No. 1481/30.05.2024).

## 3. Results

Of the 106 patients included in the study, there were 62 (58.5%) men and 44 (41.5%) women. The basic characteristics of PLHIV in the studied group are presented in Table 1.

In the studied group, DEXA showed that 87 (82%) patients had low BMD, 54 (51%) having osteopenia and 33 (31.1%) patients having osteoporosis.

Table 2 shows analysis of the BMD of 87 cases of osteopenia/osteoporosis—lombar spine BMD, femur neck BMD, distal forearm BMD.

Of the 44 evaluated women, 35 (79.6%) had osteopenia/osteoporosis and 52 (83.9%) male patients had osteopenia/osteoporosis (Table 3).

The patient’s gender was not associated with low BMD (*p* = 0.663, Chi-square test).

The median age of the patients from the studied group at the time of HIV diagnosis was 23.5 years [0:70], and at the time of DEXA scan 37 years [21:76]. The patients with osteopenia/osteoporosis were aged > 40 years with a mean age of 41.2 ± 10.2 years compared to 29.3 ± 7.3 years in patients with normal BMD (*p* = 0.0001, *t*-test).

A total of 37 patients were aged 45 years or older, including 8 patients aged 45 years and 29 patients aged ≥46 years, of whom 12 (32.4%) were women. In this age group, the patient’s gender was associated with low BMD, osteopenia and osteoporosis being more frequent among women compared to men (85.7% vs. 53.3%, *p* = 0.048, Chi-square test). All women in this subgroup were postmenopausal.

Fifteen patients (15/87, 17.2%) were diagnosed with osteopenia/osteoporosis exclusively in the distal forearm. Of these, 13 patients (86.7%) were under 40 years of age, indicating that age < 40 years was associated with forearm BMD reduction (*p* = 0.04, Chi-square test). This finding suggests that the earliest detectable changes in bone mineral density may occur in the distal forearm, possibly related to nutritional factors, vitamin D deficiency during growth, or direct effects of HIV-induced chronic inflammation and cytokine activity on bone turnover.

We found the presence of coinfection with hepatitis B virus in 20 patients, 75% of these being diagnosed with osteopenia/osteoporosis, with no statistically significant differences compared to those without coinfection (75% vs. 83.7%, *p* = 0.348, Fisher’s test).

The clinical and immunological classification for HIV infection is presented in Table 4 (CDC classification system). A total of 62 patients (62/75, 82.7%) in the AIDS stage and 25 patients (25/31, 80.1%) in the non-AIDS stage were diagnosed with osteopenia or osteoporosis. There was no significant association between HIV stage and low BMD (*p* = 1, Chi-square test).

According to BMI criteria, 52 (49.1%) patients had normal weight, 26 (24.5%) were overweight, 25 (23.6%) were obese, only 3 (2.8%) were underweight. Among the 87 PLHIV diagnosed with osteopenia/osteoporosis, 45 (51.7%) had normal weight, 22 (25.4%) were overweight, 17 (19.5%) were obese, and 3 (3.4%) were underweight. No significant association was found between BMI and osteopenia/osteoporosis (*p* = 0.884, Fisher’s test).

In our study, 40 (45.9%) patients had 25[OH]D deficiency, with an average vitamin D level of 27.7 ± 9.6 ng/mL. No significant difference in vitamin D levels was observed between patients with normal BMD and those with osteopenia/osteoporosis (27.8 ± 9.6 vs. 27.0 ± 12.6 ng/mL, *p* = 0.775, Student’s *t*-test). Vitamin D levels were similar in the two groups.

Smoking and alcohol consumption were not significantly associated with osteopenia/osteoporosis (*p* = 0.564, *p* = 0.157, Fisher’s test). Among the 39 smoking patients, 35 (89.7%) had reduced BMD compared to 52 (77.6%) of the 67 non-smokers. Similarly, 92.5% of patients who reported alcohol consumption and 81.8% of non-drinkers were diagnosed with osteopenia/osteoporosis. Smoking and alcohol consumption were not significantly associated with osteopenia/osteoporosis (*p* = 0.564 and *p* = 0.157, Fisher’s test).

A proportion of 81.1% of PLHIV had undetectable VL-HIV (<20 copies/mL) at the time of DEXA evaluation. PLHIV diagnosed with osteopenia/osteoporosis had a higher level of VL-HIV (>10,000 copies/mL), compared to PLHIV without osteopenia/osteoporosis (90% vs. 62.8%), with a mean of HIV viremia of 4.2 log10 copies/mL. Higher HIV viral load (>10,000 copies/mL) was significantly associated with osteopenia/osteoporosis (*p* = 0.018, Chi-square test).

CD4 + lymphocyte count assessment before BMD measurement showed a median value of 627 cells/mm^3^ [min 29: max 1603]. Immunological evaluation of PLHIV revealed a mean CD4 + lymphocyte count of 621.12 ± 593 cells/mm^3^ in PLHIV and osteopenia/osteopororsis and a mean CD4 + lymphocyte count of 704.93 ± 727 cells/mm^3^ in PLHIV with normal BMD, with no statistically significant differences between these two groups (*p* = 0.640, Mann–Whitney U test).

Mean CD4 lymphocyte nadir was 164.6 ± 145.7 cells/mm^3^.68 PLHIV (64.1%) in our study had nadir CD4 + count <200 cells/mm^3^. Among patients with a nadir CD4 + count <200 cells/mm^3^, 62 (91.1%) had osteopenia or osteoporosis, compared to 25 (65.8%, 25/38) of patients with nadir CD4 + ≥200 cells/mm^3^. There was a significant association between low nadir CD4 + count and reduced BMD (*p* = 0.04, Chi-square test).

Of the 83 patients who had been on ART for more than 20 years (Table 5), 76 (91.6%) had low BMD, whereas among the 23 patients on ART for less than 20 years, 11 (47.8%) had low BMD. Prolonged ART duration was significantly associated with low BMD (*p* < 0.0001, Chi-square test).

In our study, 58 patients (54.7%) used tenofovir disoproxil fumarate (TDF) and 49 patients developed osteopenia/osteoporosis, TDF containing regimens being associated with low BMD (*p* = 0.024, Chi-square test). Of the 62 patients who used protease inhibitors in their ART regimens, 53 developed osteopenia/osteoporosis, with no significant association between PIs and low BMD (*p* = 1, Fisher’s test).

## 4. Discussion

Low bone mineral density is a significant concern in people living with HIV, predisposing them to osteopenia, osteoporosis, and fragility fractures. Several factors, including HIV infection itself, antiretroviral therapy, and host characteristics, contribute to accelerated bone loss in this population. This study evaluated BMD in patients with HIV and identified associated risk factors.

In our cohort, osteoporosis was detected in 31.1% of patients, a prevalence higher than that reported in several other studies. This may be explained by the long duration of ART exposure in many patients (83% received ART for more than 20 years), the frequent use of TDF-based regimens, and the unique epidemiological features of the Romanian HIV population, with many individuals infected during childhood in the 1980s. In addition, the relatively high proportion of postmenopausal women in our study group may have contributed to the increased prevalence. These findings emphasize the importance of early screening and monitoring of bone health in this population.

We did not observe significant associations between low BMD and BMI, vitamin D levels, smoking, or alcohol use. This may be due to the relatively small sample size, limited variability in these parameters, and the stronger impact of HIV-related and ART-related factors in our cohort. Another similar study that was conducted in Istanbul, Turkey in 2024, examined 116 PLHIV. Among them, 47.4% were diagnosed with either osteopenia or osteoporosis, with 4.3% having osteoporosis and 43.1% presenting osteopenia. Patients with these bone conditions tended to be older (39.2 ± 11.0 years vs. 32.0 ± 8.6 years, *p* = 0.0001), had lower vitamin D levels (16.0 ± 5.0 vs. 24.4 ± 6.3, *p* = 0.0001), lower BMI (23.0 ± 4.0 vs. 24.6 ± 4.6, *p* < 0.05), and lower CD4 counts (405.1 ± 885.0 vs. 467.3 ± 695.1). Additionally, 41.8% had CD4 counts ≤200/µL, compared to 18.0% in the non-osteopenia/osteoporosis group (*p* = 0.005). No significant differences were found regarding gender distribution, smoking, alcohol and drug use, comorbidities, additional medication usage, or HIV RNA levels exceeding 100,000 copies/mL. Multivariate analysis identified age and vitamin D levels as significant independent risk factors for osteoporosis and osteopenia (*p* < 0.05) [17].

Regarding ART regimens, our findings confirmed the association between TDF-containing regimens and reduced BMD. Recent studies suggest that tenofovir alafenamide (TAF) has a safer profile for bone and renal health compared to TDF, but most of our patients were treated with TDF and very few had switched to TAF. This difference may partly explain the high prevalence of bone demineralization observed in our study [18]. Several studies demonstrated that ART impacts bone, showing an association between TDF and reduced BMD [19,20]. In a prospective study involving treatment-naive people with HIV, participants were assigned to one of three ARV regimens: TDF, TAF, or abacavir based therapies. TDF treatment was linked to more pronounced bone deterioration at both 12 and 48 weeks [5]. Another study performed in Chonburi, Thailand between November 2010 and May 2014, included 106 PLHIV (75 and 31 started TDF- and non-TDF-containing regimen, respectively) and 66 HIV-uninfected individuals. The mean percent changes in BMD were significantly different between TDF and non-TDF users [21].

A 2025 longitudinal study in the UK assessed BMD in 130 young individuals aged 15–25 with perinatally acquired HIV. The study found that low BMD was prevalent and associated with factors such as age, ART regimen, and immune suppression. Notably, a two-year follow-up indicated that transitioning from TDF to TAF was linked to improved BMD outcomes [22].

An unexpected finding in our study was the association of low forearm BMD with age < 40 years, which contrasts with the typical pattern of osteoporosis, where bone loss is usually more pronounced in older adults. One possible explanation is that the distal forearm, composed mainly of cortical bone, may reveal early site-specific skeletal changes in patients with HIV, before generalized bone loss becomes evident at other skeletal sites such as the spine or hip. Some other factors may contribute to this observation:

1. Differential ART Exposure: Younger patients may have been exposed to TDF or other regimens known to affect bone health early in treatment, leading to site-specific bone loss in the forearm.
2. Immune Status: Younger individuals with a history of low CD4 nadir might experience more pronounced immune-related bone resorption despite their age.
3. Lifestyle and Nutritional Factors: Variations in physical activity, calcium intake, or vitamin D status among younger participants may influence forearm BMD differently than lumbar or hip sites.
4. Site-specific Vulnerability: Peripheral bones like the forearm may respond differently to systemic factors such as inflammation, ART, or hormonal changes, which could explain why younger patients present altered forearm BMD while other sites are relatively preserved.

Similar associations have been described in other cohorts of young adults living with HIV, where early bone demineralization was reported at peripheral sites and linked to both HIV infection itself and antiretroviral exposure [23].

We emphasize the need for re-evaluation of patients with borderline or altered forearm BMD values. Periodic monitoring of bone mineral density in these individuals can help identify early progression of bone loss and guide timely interventions to prevent osteoporosis and fractures. This targeted re-evaluation is particularly important in patients with additional risk factors, such as low CD4 nadir or long-term ART.

Another study performed in Turkey between June 2010 and May 2011 included 126 patients with HIV, that had been evaluated with DEXA. Median age was 40.1 years; 84% were male; 35.7% patients had AIDS, 63.5% were treated with ART. Osteopenia and osteoporosis were diagnosed in 53.9% and 23.8%, respectively. Mean plasma VL-HIV was 5.2 log10 copies/mL and CD4 lymphocyte nadir was 313.8 cells/mm^3^. Factors associated with BMD were high VL-HIV, using and duration of ART, with no association between sex and osteopenia/osteoporosis. Males showed higher rates of osteoporosis than females [24].

A cross-sectional study was conducted among PLHIV and receiving ART at the largest tertiary adult teaching *Hosp.* in Lusaka, Zambia, between 1 August 2019, and 31 December 2020. The study included participants aged 50 to 69 years. BMD was assessed DEXA. Among the 315 participants, 43.8% were female, and the median age was 55 years. The overall prevalence of low BMD was 82.6%, with 34.0% of participants diagnosed with osteopenia and 48.6% with osteoporosis. Age ≥55 years was independently associated with increased odds of osteoporosis (*p* < 0.001). A CD4 count ≥500 cells/mm^3^ (*p* < 0.001) and higher BMI (*p* = 0.008) were associated with reduced odds of osteoporosis [25].

Numerous studies have documented the widespread occurrence of bone demineralization among both younger and older HIV-positive patients. This decline in BMD is closely linked to an increased likelihood of bone fractures. HIV infection has several general effects that can contribute to a lower BMD. These risk factors include low BMI, physical inactivity, malabsorption—particularly of calcium—hypogonadism, vitamin D deficiency, as well as smoking, and alcohol abuse [20,21,26].

Age >40 years was identified as an independent risk factor for osteoporosis in our study, which is consistent with findings from other studies highlighting the role of aging in the pathogenesis of bone loss among people living with HIV. As individuals age, there is a natural decline in bone density; however, the combination of HIV infection and ART appears to exacerbate this process. This age-related effect may be compounded by other factors such as decreased physical activity, changes in diet, and other comorbidities prevalent in older populations.

In addition to age, high VL-HIV has been identified as an independent risk factor for low BMD. Chronic high levels of VL-HIV contribute to persistent systemic inflammation, which is thought to disrupt the delicate balance between bone formation and resorption. Elevated HIV viral load is also linked to higher levels of pro-inflammatory cytokines, which promote osteoclast activity and enhance bone breakdown, further increasing the risk of osteoporosis and fractures.

The nadir CD4 + count, defined as the lowest recorded CD4 + T-cell count during the course of HIV infection, is another important predictor of low BMD. In the study we performed, a nadir CD4 + count of less than 200 cells/mm^3^ was associated with a higher risk of bone loss, likely due to prolonged periods of immune suppression, which not only affects bone metabolism but also reduces the body’s ability to repair and maintain bone tissue. Individuals with a low nadir CD4 + count are also more likely to experience a greater degree of immune activation, which has been implicated in the pathogenesis of bone loss in HIV.

A 2025 cross-sectional study conducted in Iran assessed BMD in patients aged 30–50 years undergoing ART for at least six months. The study identified a strong association between lower CD4 nadir and reduced BMD, particularly in the lumbar spine region. This association remained significant even after adjusting for other known risk factors such as age, body mass index, and duration of ART. These findings suggest that a lower CD4 nadir may contribute to increased bone loss in this population, highlighting the importance of monitoring CD4 nadir as a potential predictor of bone health [27].

Postmenopausal women living with HIV are at an elevated risk of bone fractures, owing to the combined effects of HIV-related factors and the physiological changes associated with menopause. Across 223 relevant studies, evidence consistently demonstrates that postmenopausal women living with HIV exhibit significantly lower BMD and higher rates of fracture when compared with both HIV-negative postmenopausal women and premenopausal HIV-positive women [28]. Ahmed et al. demonstrated that women living with HIV are at increased risk of experiencing early menopause, a factor that may contribute to a heightened vulnerability to osteoporosis [29].

In our study, osteopenia and osteoporosis were frequently observed among postmenopausal women living with HIV, aged over 45 years, suggesting a synergistic effect between HIV infection, the aging process, and hormonal changes associated with menopause.

In Romania, a cross-sectional study was performed in “Prof. Dr. Matei Balş” National Institute for Infectious Diseases, Bucharest between 2016 and 2018. The study included 97 HIV-positive patients with the median age of 26 years. According to the DEXA T-scores, 10 males and 8 females had osteopenia and 4 males and 4 females had osteoporosis. In males, low T-scores were associated with decreased BMI, low vitamin-D levels and high VL-HIV [30].

Given the high prevalence of bone mineral density alterations, conducting osteodensitometry measurements for patients with HIV would be beneficial. In this population, both conventional risk factors for reduced BMD and those specifically linked to HIV infection play a significant role, warranting greater focus and careful management.

A meta-analysis indicates that PLHIV experience a 35% increased risk of fragility fractures starting as early as 40 years of age. Additionally, a registry study found that PLHIV face a threefold, ninefold, and ninefold greater risk of experiencing fractures at any site, the hip, and the spine, respectively [31,32].

Given these multiple and overlapping risk factors, it is essential to implement early screening for low BMD in PLHIV, especially those over 40 years of age, with a history of high viral load, low nadir CD4 + count, and prolonged ART exposure. Interventions, including lifestyle modifications, calcium and vitamin D supplementation, and the possible adjustment of ART regimens, particularly replacing TDF with TAF, should be considered to mitigate the risk of osteoporosis and related fractures in this vulnerable population. Early identification and proactive management of bone health can significantly reduce the burden of fragility fractures, improving the long-term health outcomes of PLHIV.

## 5. Conclusions

This study highlights a high prevalence of bone mass reduction among patients with HIV from CRC.

In our study, osteoporosis and osteopenia were associated with age > 40 years, higher levels of VL-HIV ( > 10000 copies/mL), nadir CD4 + level <200 cells/mm^3^, prolonged ART exposure and TDF containing regimens. Also, osteopenia and osteoporosis were more frequently observed in postmenopausal women living with HIV, aged over 45 years old.

Low BMD at the forearm level was correlated with age < 40 years, suggesting the need to include forearm bone density assessment as part of routine osteopenia/osteoporosis screening in PLHIV.

The limitations of our study include the small sample size and the absence of longitudinal monitoring of the patients. Additionally, while DEXA is a standard method for measuring BMD, it does not assess bone quality, and other factors such as bone turnover markers and fracture risk were not evaluated. Future longitudinal studies that incorporate these factors could provide a more comprehensive understanding of bone health in HIV patients. Advanced imaging techniques (HR-pQCT) and biochemical markers of bone turnover (e.g., osteocalcin) were not available due to resource limitations, representing an additional limitation.

Bone mineral density remains a critical aspect of health management for individuals living with HIV, particularly for those on long-term ART regimens. Clinicians should be vigilant in monitoring bone health, particularly in patients aged over 40 years old, those with lower CD4 counts, and those on TDF-based ART. Interventions aimed at preserving bone health, such as calcium and vitamin D supplementation, weight-bearing exercises, and adjustments in ART regimens, may help mitigate the risk of osteoporosis and fractures in this vulnerable population.

These findings support the inclusion of routine bone health monitoring in the standard care of patients with HIV, as well as the need for reevaluation. Early detection of osteopenia and osteoporosis enables timely intervention, reduces the risk of fractures, and improves long-term quality of life. Integrating bone health assessments into routine care supports a more comprehensive and preventive approach to managing the long-term effects of HIV and its treatment.

## Figures and Tables

**Table 1:** Basic characteristics of PLHIV.

Basic Characteristics			Number of Patients (%)
Patient’s age (years old)	Mean ± SD 41.1 ± 10.5	18–25 26–35 36–45 ≥46	6 (5.7%) 34 (32%) 37 (34.9%) 29 (27.4%)
Place of residence	Urban Rural		67 (63.2%) 39 (36.8%)
Probable route of HIV transmission	Parenteral Sexual Mother-to-child		42 (39.6%) 62 (58.5%) 2 (1.9%)
Co-infections with hepatitis viruses	Hepatitis B Hepatitis B + D No		19 (18%) 1 (0.9%) 86 (81.1%)
Smoking	Yes No		39 (36.8%) 67 (63.2%)
Alcohol consumption	Yes No		40 (37.7%) 66 (62.3%)

**Table 2:** Analysis of the BMD.

DEXA Evaluation	Number of Patients	Mean ± SD	Minimum	Maximum
Age	87	41 ± 10.2	21	68
Lombar spine T-score L1	87	−0.6 ± 1.7	−3.7	7.7
Lombar spine T-score L2	87	−1 ± 1.7	−7	4.8
Lombar spine T-score L3	87	−0.7 ± 1.9	−7.8	6
Lombar spine T-score L4	87	−0.4 ± 1.8	−5.8	5
Femur neck T-score	87	−0.5 ± 1	−3.1	2
Distal forearm T-score	87	−1.6 ± 1.4	−4.8	1.4

**Table 3:** Distribution of osteopenia/osteoporosis in the studied group.

	Number of Patients	Male (%)	Female (%)
Osteopenia	54	31 (50%)	23 (52.3%)
Osteoporosis	33	21 (33.9%)	12 (27.3%)
Normal BMD	19	10 (16.1%)	9 (20.4%)
Total	106	62 (58.5%)	44 (41.5%)

**Table 4:** Stage of HIV infection.

Stage Of HIV Infection	Number of Patients (%)
A1	1 (0.9%)
B1	2 (1.9%)
C1	1 (0.9%)
A2	9 (8.5%)
B2	19 (18%)
C2	7 (6.6%)
A3	0 (0%)
B3	30 (28.3%)
C3	37 (34.9%)

**Table 5:** Duration of ART.

Duration of ART	Number of Patients (%)
1–4 years	3 (2.8%)
5–10 years	6 (5.7%)
11—20 years	14 (13.2%)
> 20 years	83 (78.3%)

## Data Availability

The original contributions presented in this study are included in the article. Further inquiries can be directed to the corresponding author.

## References

[1] https://www.who.int/teams/global-hiv-hepatitis-and-stis-programmes/hiv/strategic-information/hiv-data-and-statistics.

[2] https://www.cnlas.ro/index.php/date-statistice.

[3] (2006). Life Doesn’t Wait. https://www.hrw.org/report/2006/08/01/life-doesnt-wait/romanias-failure-protect-and-support-children-and-youth-living.

[4] Preda M., Manolescu L.C.S. (2022). Romania, a Harbour of HIV-1 Subtype F1: Where Are We after 33 Years of HIV-1 Infection?. Viruses.

[5] Lima A. (2011). Osteopenia and osteoporosis in people living with HIV: multiprofessional approach. HIV/AIDS-Res. Palliat. Care.

[6] Hasan S., Aqil M., Panigrahi R. (2022). HIV-Associated Systemic Sclerosis: Literature Review and a Rare Case Report. Int. J. Environ. Res. Public Heal..

[7] Pramukti I., Lindayani L., Chen Y.-C., Yeh C.-Y., Tai T.-W., Fetzer S., Ko N.-Y. (2020). Bone fracture among people living with HIV: A systematic review and meta-regression of prevalence, incidence, and risk factors. PLOS ONE.

[8] Hansen A.-B.E., Gerstoft J., Kronborg G., Larsen C.S., Pedersen C., Pedersen G., Obel N. (2012). Incidence of low and high-energy fractures in persons with and without HIV infection. AIDS.

[9] Cummins N.W. (2022). Metabolic Complications of Chronic HIV Infection: A Narrative Review. Pathogens.

[10] E Sax P., Wohl D., Yin M.T., Post F., DeJesus E., Saag M., Pozniak A., Thompson M., Podzamczer D., Molina J.M. (2015). Tenofovir alafenamide versus tenofovir disoproxil fumarate, coformulated with elvitegravir, cobicistat, and emtricitabine, for initial treatment of HIV-1 infection: Two randomised, double-blind, phase 3, non-inferiority trials. Lancet.

[11] Hileman C.O., Eckard A.R., McComsey G.A. (2015). Bone loss in HIV. Curr. Opin. Endocrinol. Diabetes.

[12] Gibellini D., De Crignis E., Ponti C., Cimatti L., Borderi M., Tschon M., Giardino R., Re M.C. (2008). HIV-1 triggers apoptosis in primary osteoblasts and HOBIT cells through TNFα activation. J. Med Virol..

[13] Azarboo A., Hemmatabadi M., Fahimfar N., Faghihi Z., SeyedAlinaghi S., Shirzad N., Abbasian L. (2025). Evaluation of bone mineral density and its influencing factors in patients infected with HIV under antiretroviral therapy. BMC Infect. Dis..

[14] Sözen T., Özışık L., Başaran N.Ç. (2019). An overview and management of osteoporosis. Eur. J. Rheumatol..

[15] Williams S., Khan L., Licata A.A. (2021). DXA and clinical challenges of fracture risk assessment in primary care. Clevel. Clin. J. Med..

[16] https://www.who.int/data/gho/data/themes/topics/topic-details/GHO/body-mass-index.

[17] Han W.M., Wattanachanya L., Apornpong T., Jantrapakde J., Avihingsanon A., Kerr S.J., Teeratakulpisarn N., Jadwattanakul T., Chaiwatanarat T., Buranasupkajorn P. (2020). Bone mineral density changes among people living with HIV who have started with TDF-containing regimen: A five-year prospective study. PLOS ONE.

[18] Maggiolo F., Rizzardini G., Raffi F., Pulido F., Mateo-Garcia M.G., Molina J.-M., Ong E., Shao Y., Piontkowsky D., Das M. (2019). Bone mineral density in virologically suppressed people aged 60 years or older with HIV-1 switching from a regimen containing tenofovir disoproxil fumarate to an elvitegravir, cobicistat, emtricitabine, and tenofovir alafenamide single-tablet regimen: A multicentre, open-label, phase 3b, randomised trial. Lancet HIV.

[19] Cazanave C., Dupon M., Lavignolle-Aurillac V., Barthe N., Lawson-Ayayi S., Mehsen N., Mercié P., Morlat P., Thiébaut R., Dabis F. (2008). Reduced bone mineral density in HIV-infected patients: Prevalence and associated factors. AIDS.

[20] Jones S., Restrepo D., Kasowitz A., Korenstein D., Wallenstein S., Schneider A., Keller M.J. (2007). Risk factors for decreased bone density and effects of HIV on bone in the elderly. Osteoporos. Int..

[21] Duvivier C., Kolta S., Assoumou L., Ghosn J., Rozenberg S., Murphy R.L., Katlama C., Costagliola D. (2009). Greater decrease in bone mineral density with protease inhibitor regimens compared with nonnucleoside reverse transcriptase inhibitor regimens in HIV-1 infected naive patients. AIDS.

[22] Henderson M., Blenkinsop A., Ratmann O., Cheung M., Lyall H., Fidler S., Foster C., The BONDY study group (2025). Bone health in a U.K. cohort of youth living with perinatally acquired HIV‐1: A longitudinal study. J. Int. AIDS Soc..

[23] https://www.nature.com/articles/ncomms9282.

[24] Aydın O.A., Karaosmanoglu H.K., Karahasanoglu R., Tahmaz M., Nazlıcan O. (2013). Prevalence and risk factors of osteopenia/osteoporosis in Turkish HIV/AIDS patients. Braz. J. Infect. Dis..

[25] Sun C.P., Shanty V., Wong P.S. (2024). PREVALENCE AND ASSOCIATED RISK FACTORS OF LOW BONE MINERAL DENSITY IN MALAYSIANS LIVING WITH HIV. J. ASEAN Fed. Endocr. Soc..

[26] Bonjoch A., Figueras M., Estany C., Perez-Alvarez N., Rosales J., del Rio L., di Gregorio S., Puig J., Gómez G., Clotet B. (2010). High prevalence of and progression to low bone mineral density in HIV-infected patients: A longitudinal cohort study. AIDS.

[27] Zhang J., Ma B., Han X., Ding S., Li Y. (2022). Global, regional, and national burdens of HIV and other sexually transmitted infections in adolescents and young adults aged 10–24 years from 1990 to 2019: a trend analysis based on the Global Burden of Disease Study 2019. Lancet Child Adolesc. Heal..

[28] Jamshaid M., Heidari A., Hassan A., Mital D., Pearce O., Panourgia M., Ahmed M.H. (2024). Bone Loss and Fractures in Post-Menopausal Women Living with HIV: A Narrative Review. Pathogens.

[29] Ahmed M.H., Bondje S., Jiwan R., Rawther F., Duku A., Husain N.E., Woodward C., Mital D. (2021). Early menopause in acquired immunodeficiency syndrome. J. Res. Med Sci..

[30] Negru A. (2019). Bone Quality in a Young Cohort of Hiv-Positive Patients. Acta Endocrinol..

[31] Shiau S., Broun E.C., Arpadi S.M., Yin M.T. (2013). Incident fractures in HIV-infected individuals. AIDS.

[32] Prieto-Alhambra D.M., Güerri-Fernández R., De Vries F., Lalmohamed A.M., Bazelier M., Starup-Linde J., Diez-Perez A., Cooper C.M., Vestergaard P. (2014). HIV Infection and Its Association With an Excess Risk of Clinical Fractures. Am. J. Ther..

